# Possible Ovarian and Peritoneal Carcinoma Presenting in a Mediastinal Lymph Node and Pleural Effusion: A Case Report and Review of the Literature

**DOI:** 10.7759/cureus.44564

**Published:** 2023-09-02

**Authors:** Keana-Kelley D Swanner, Nick W Lanpher, Aasim Sehbai

**Affiliations:** 1 College of Medicine, Alabama College of Osteopathic Medicine, Dothan, USA; 2 Hematology and Oncology, Alabama Cancer Care, Anniston, USA

**Keywords:** elevated ca-125, mediastinal lymph node, malignant plural effusion, ovarian cancers, ovarian carcinoma

## Abstract

Ovarian carcinoma often doesn't show noticeable symptoms and is frequently diagnosed at an advanced stage. It is the most fatal cancer within the gynecologic system. Our understanding of ovarian pathology is limited, necessitating the use of multiple markers to accurately detect ovarian cancer, particularly when it presents abnormally, such as in pleural effusion or lymph nodes.

A 45-year-old woman presented to the emergency room (ER) due to abdominal pain lasting for two weeks. A computed tomography (CT) scan revealed peritoneal carcinomatosis accompanied by ascites and calcification in the lymph nodes. The likely primary sources were determined to be mucinous adenocarcinomas from either the colon or ovary. Following the CT findings, a fine needle aspiration was conducted on a perigastric lymph node. Histopathology results indicated a "poorly differentiated carcinoma [with] malignant cells present." Subsequently, a PowerPort was inserted, and adjuvant chemotherapy commenced two days later, utilizing a combination of carboplatin, bevacizumab, and paclitaxel. Paracentesis was performed, yielding clear-yellow fluid. However, abdominal fullness gradually increased again after paracentesis. The patient began experiencing more intense abdominal pain, particularly in the left lower quadrant. Surgical exploration revealed widespread disease involvement throughout the intestines.

Our patient exhibited an atypical manifestation of ovarian carcinoma, challenging its identification due to ectopic foci and the absence of many distinctly identifiable markers. Through comprehensive testing and a process of elimination, we successfully differentiated ovarian carcinoma from other potential cancers. The conclusive histopathological report, along with a markedly elevated CA-125 level, provided substantial support for the probable final diagnosis of ovarian carcinoma.

Despite numerous advancements in staining and identification techniques, the diagnosis of ovarian carcinoma remains inadequately understood. Identifying ovarian carcinoma without clear visualization is often challenging, and further research is warranted to enhance our understanding of pathological methods. Moreover, there is a need to prioritize the development and exploration of ovarian carcinoma screening and testing methods to prevent delayed disease detection.

## Introduction

Ovarian carcinoma has limited subjective symptoms, and a substantial number of cases may be found late in the disease course. When ovarian carcinomas present in stages III or IV, it is indicative of a poor prognosis. Ovarian carcinomas diagnosed at stage IV have a 17% five-year survival rate [1]. Currently, the lack of screening techniques and the late onset of symptoms lead to a high mortality rate. Additionally, ovarian carcinoma continues to be one of the leading causes of cancer deaths in women. This case presents a rare presentation of a possible ovarian carcinoma that was incidentally discovered through a pleural effusion. Abundant pathological testing has led to this diagnosis and is discussed here to aid in the accumulation of knowledge that can lead to better screening and earlier detection of ovarian carcinomas in the future.

## Case presentation

On October 5, 2022, a 45-year-old Caucasian female arrived at the emergency room (ER), reporting dull abdominal pain and bloating that had persisted for the past two weeks. The patient described her abdomen as swollen and distended in comparison to her usual baseline. She also observed fresh, bright red blood in her diarrhea, which she attributed to hemorrhoids. She endorsed some diarrhea and constipation but denied additional gastrointestinal symptoms. Her most recent menstrual cycle occurred approximately one year prior. The patient denied using drugs, alcohol, or tobacco.

**Figure 1:**
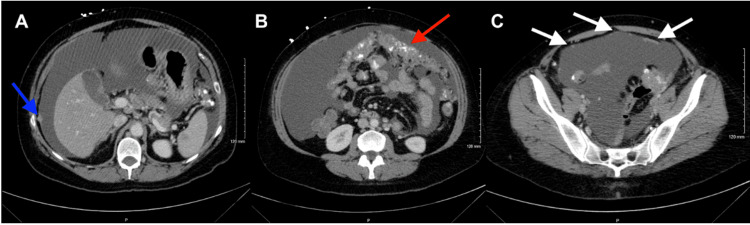
A computed tomography (CT) scan of the abdomen and pelvis was performed on October 5, 2022. A: Evidence of a discrete nodule (blue arrow) in the peritoneum suggests peritoneal carcinomatosis. B: Omental caking (red arrow) with associated calcification of the mesentery suggests adenocarcinoma. C: Anterior peritoneal lining demonstrates nodular enhancement and thickening (white arrows), further indicating carcinomatosis. Ascites is seen throughout the CT images provided, specifically laterally and posteriorly in Figure 1A and B (black fluid within the abdominal cavity).

A computed tomography (CT) scan of the abdomen and pelvis was performed on October 5 (Figure 1). It revealed peritoneal carcinomatosis accompanied by ascites and calcification in the lymph nodes. The likely primary sources were determined to be mucinous adenocarcinomas from either the colon or ovary. There was also questionable gastric antral thickening. No significant ovarian or uterus findings were present on the CT scan.

**Figure 2:**
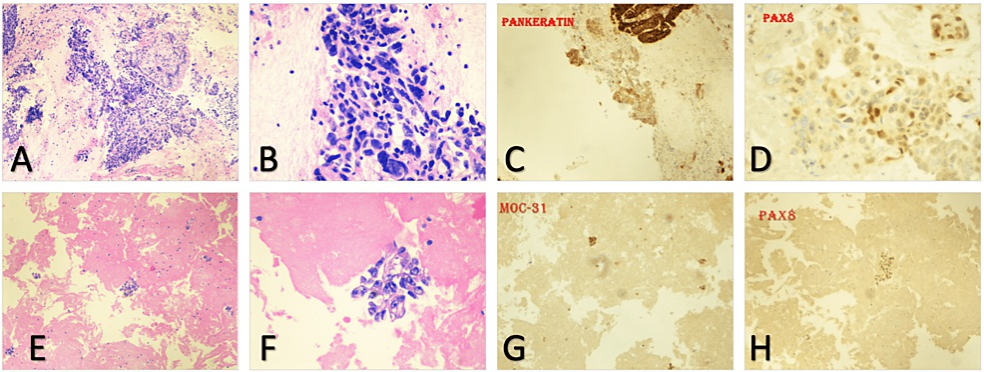
Peritoneal fluid and Peri-gastric lymph node staining A-D: Fine needle aspiration performed on a perigastric lymph node E-H: Ascitic fluid histopathology A, B, E, and F utilized H&E stains for mitotic activity; the remaining stains are indicated on their respective slides.

Fine needle aspiration was conducted on a perigastric lymph node following CT findings (Figure 2). Histopathology analysis revealed a "poorly differentiated carcinoma [with] malignant cells present," identified through the following immunohistochemical stains: CK7(+)/CK20(+), Moc 31(+), PAX8(+), P16(+), RCC(-), ER(-), P63-, GATA3(-), and TTF-1(-).

The subsequent day, ascitic fluid obtained post-paracentesis was examined. Histopathology results indicated Moc31(+)/Ber-EP4(+), PAX8(+), ER(-), Calretinin(-), CDX2(-), CD10(-)/RCC(-), leading to the diagnosis of metastatic carcinoma of mullerian/genitourinary origin (Figure 2). HER2 status was non-amplified on in-situ hybridization. This led to a potential diagnosis of a malignant neoplasm of an unspecified ovary and a secondary malignant neoplasm of the retroperitoneum and peritoneum, along with malignant ascites.

The carcinoembryonic antigen (CEA) level was reported as <0.5 ng/mL, within the normal range of 0.0-3.0 ng/mL. On October 18, CA-125 was measured at 4686 U/mL. The final histopathological report, combined with the significantly elevated CA-125 level, strongly supported the most probable diagnosis of ovarian carcinoma.

Placement of PowerPort was done on October 17, and adjuvant chemotherapy began two days later with a combination of carboplatin, bevacizumab, and paclitaxel. Paracentesis was performed on October 19, and 2700 cc of clear-yellow fluid was removed. The patient appeared to have less pain and more stability, and she tolerated chemotherapy without complications.

Following the paracentesis procedure, there was a gradual increase in abdominal fullness. Subsequently, the patient began to experience more intense abdominal pain, specifically localized to the left lower quadrant. The patient attributed this discomfort to constipation and was prescribed a bowel preparation in an attempt to alleviate it. Despite these efforts, the patient's condition continued to deteriorate, prompting a consultation with the gastroenterology team.

**Figure 3:**
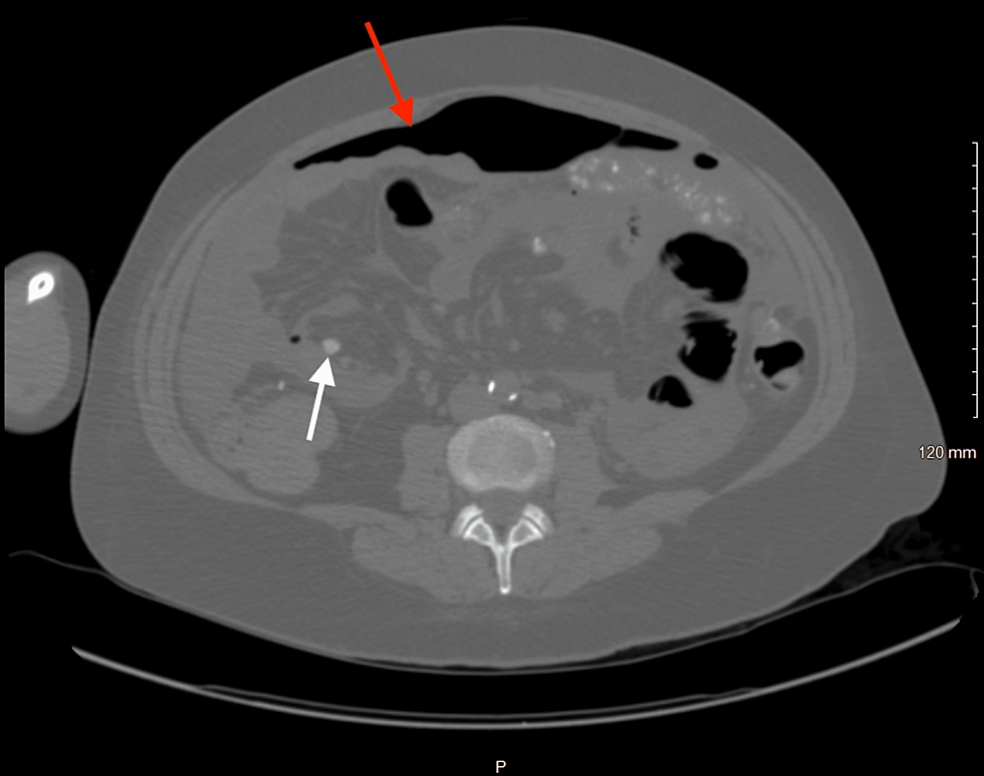
A CT of the abdomen and pelvis without IV contrast was performed on October 25, 2022 Impression noted bibasilar discoid atelectasis and/or atelectatic infiltrates. A moderate amount of pneumoperitoneum (red arrow) is worrisome for a ruptured viscus; it is of note that the patient’s last paracentesis was seven days ago. Moderate abdominal and pelvic ascites. Redemonstration of findings compatible with extensive peritoneal carcinomatosis (white arrow).

On October 25, a CT scan of the abdomen and pelvis without IV contrast (Figure 3) was conducted. The scan revealed a moderate amount of pneumoperitoneum, raising concerns about a potential ruptured viscus. Additionally, there was the presence of moderate abdominal and pelvic ascites, along with a recurrence of findings consistent with extensive peritoneal carcinomatosis.

Given these new developments, the general surgery team was urgently consulted due to the emergence of a moderate-to-large pneumoperitoneum.

The patient was therefore taken into surgery for exploratory laparotomy and possible surgical excision, but the infiltration of carcinomatosis was far more extensive than initially thought. On October 26, 2022, the patient was discharged from the hospital and placed on home hospice care to be with her family.

## Discussion

Ovarian carcinoma ranks as the fifth leading cause of cancer-related death in women, and it stands as the second most prevalent gynecological malignancy overall [2]. Presently, the cure rate for ovarian carcinoma rests at approximately 30%, largely owing to its transient chemosensitivity [3]. A heightened level of suspicion becomes essential during the assessment of nonspecific abdominal symptoms, even in the absence of a detectable adnexal mass on CT imaging. The initial serendipitous discovery of pleural effusion prompted a subsequent investigative process that entailed extensive immunohistochemical staining, ultimately culminating in a definitive diagnosis.

The diagnosis in this case was achieved through extensive histopathological staining. The uncertainty surrounding the origin of malignancy necessitated the use of various immunohistochemical stains to either confirm or eliminate different tissue sources. Given the location of the suspected malignancy, multiple stains were essential to accurately detect atypical cells.

A positive Moc31 stain indicates carcinoma and helps distinguish cells of mesothelioma origin. This differentiation is crucial, particularly when testing ascitic fluid, to ensure that the sample does not originate from the typical abdominal lining [4]. Ber-EP4 staining identifies epithelial cell adhesion molecules, similarly pinpointing epithelial or carcinoma origin while ruling out mesothelioma. Additionally, calretinin identifies mesothelioma origin in pleural and peritoneal specimens [5, 6].

Collectively, the utilization of these three stains, namely Moc31(+), Ber-EP4(+), and Calretinin(-), provides compelling evidence of carcinoma.

Upon confirming carcinoma, further efforts were undertaken to ascertain its origin. Given the patient's age and the elevated risk of breast cancer in the US, staining for estrogen receptors (ER) was conducted, yielding a negative result [7]. Furthermore, the possibility of gastrointestinal pathology was dismissed through CDX2 negative staining, a highly sensitive and specific marker for intestinal-origin adenocarcinoma [8].

The positive PAX8 stain emerged as a pivotal distinguishing factor, signifying Müllerian origin. This finding, when coupled with the patient's clinical presentation, strongly suggests ovarian pathology [9]. With Müllerian origin established, the subsequent objective was to assess any potential renal involvement. This assessment was accomplished through CD10 and RCC staining, both of which indicate renal cell carcinoma and clear cell origins. However, both stains yielded negative results, leading to the diagnosis of metastatic carcinoma originating from the genitourinary/Müllerian tract [10].

This diagnosis, in conjunction with a CA125 level significantly surpassing normal parameters, strongly indicated the likelihood of metastatic ovarian carcinoma. A comprehensive discussion of immunohistochemical staining underscores the pivotal role cytology plays in identifying the primary source of peritoneal metastatic cancer, particularly in cases involving vague patient symptoms and the absence of evident primary masses. Furthermore, cytology is indispensable for tailoring cancer therapies, as they are often highly specialized.

Significant progress has been made in advancing our comprehension of ovarian carcinoma pathogenesis. Ovarian carcinoma can be broadly divided into two main categories: type I, characterized by low-grade tumors with limited malignant potential, and type II, characterized by high-grade tumors with an increasing likelihood of metastasis [11]. Type I tumors follow a stepwise progression from dysplasia to neoplasia and are noteworthy for their diagnosis at a younger age, often correlating with improved overall survival. In contrast, type II tumors lack identifiable precursor morphological changes and are predominantly identified in postmenopausal women, accompanied by a significant reduction in median overall survival [12]. A distinctive aspect of our case is that our patient, at 45 years old, had just met the criteria for menopause, having experienced her last menstrual cycle only a year prior. Her ovarian carcinoma had widely metastasized despite her age and symptoms, with her presentation being overshadowed by a previously diagnosed case of irritable bowel syndrome (IBS).

Ovarian carcinoma, particularly of the type II variety, demonstrates a propensity for more facile peritoneal metastasis when compared to other metastatic malignancies. The ovaries, positioned within the peritoneal fluid, serve as a conduit for metastatic spread across the peritoneum or omentum, a phenomenon well exemplified by omental caking and peritoneal carcinomatosis [3, 13]. Similarly, the question arises regarding the timing of ascites onset, whether it precedes metastasis or results from it. Nevertheless, ascites have been established as an indicator of more advanced and voluminous disease [14].

In our case, the combination of a late presentation and the vagueness of symptoms led to an unfavorable outcome. It is crucial to enhance screening efforts in women and refine the clinical diagnostic algorithm for ovarian carcinomas to ultimately improve the prognosis. During the laparotomy exploration, the disease had advanced beyond the scope of surgical excision, leaving the patient and her family without a curative option.

Malignant pleural effusion remains a prevalent complication of recurrent or advanced ovarian carcinoma; however, the accuracy of cytologic testing for this condition remains limited [15]. Research should be conducted to enhance the cytology screening for malignant cells in pleural effusions, aiming to augment diagnostic tests and enhance prognostic factors for future patients. Moreover, it is essential to explore potential treatment approaches given the relatively low cure rate of ovarian carcinoma. Detailed investigations comparing surgical versus chemotherapeutic methods need to be pursued within the realm of gynecologic cancers.

Moreover, in cases where the indicators of malignancy remain unclear, fostering a heightened clinical suspicion for potential endometrial or ovarian pathologies could result in prompt detection and a more favorable opportunity for treatment. The recent advancements that have led to a novel diagnostic approach for distinguishing between type I and type II carcinomas of the ovary should have been swiftly employed in this patient's case and should be adopted for future patients as well. This approach ensures a personalized strategy for both diagnosis and treatment, ultimately leading to improved outcomes for future patients.

This may encompass, for instance, the utilization of BRCA1 and BRCA2 testing, which have been associated with 90% of hereditary ovarian carcinoma cases. Additionally, at the molecular level, high-grade serous carcinomas often exhibit TP53 gene mutations in nearly 80% of cases, along with a high Ki67 proliferation index ranging between 50% and 75%. Overexpression of HER2/neu is also evident in 20-67% of cases, while AKT activation is found in 12-30%, and p16 inactivation occurs in 15% of cases [16]. While some of these markers were employed in diagnosing this patient, the diagnostic approach can be more broadly adopted in the future.

## Conclusions

This particular case, potentially involving ovarian and peritoneal carcinomas, serves as a prominent illustration of the existing constraints in screening and detecting ovarian carcinomas. Despite extensive pathological examinations, the underlying pathogenesis of this carcinoma remains elusive, and physicians encountered challenges in distinguishing the metastasis and formulating targeted treatment. This uncommon presentation warrants consideration when evaluating a patient with peritoneal effusions and non-specific drainage. It underscores the necessity for additional detection methods to establish future diagnoses and treatment strategies, preventing the progression of cases in a similar manner.
